# Filamentation protects *Candida albicans* from amphotericin B-induced programmed cell death via a mechanism involving the yeast metacaspase, *MCA1*

**DOI:** 10.15698/mic2016.07.512

**Published:** 2016-04-25

**Authors:** David J. Laprade, Melissa S. Brown, Morgan L. McCarthy, James J. Ritch, Nicanor Austriaco

**Affiliations:** 1 Department of Biology, Providence College, 1 Cunningham Square, Providence, Rhode Island 02918, U.S.A.

**Keywords:** Candida albicans, amphotericin B, caspofungin, MCA1, programmed cell death, filamentation

## Abstract

The budding yeast *Candida albicans* is one of the most significant fungal pathogens worldwide. It proliferates in two distinct cell types: blastopores and filaments. Only cells that are able to transform from one cell type into the other are virulent in mouse disease models. Programmed cell death is a controlled form of cell suicide that occurs when *C. albicans* cells are exposed to fungicidal drugs like amphotericin B and caspofungin, and to other stressful conditions. We now provide evidence that suggests that programmed cell death is cell-type specific in yeast: Filamentous *C. albicans* cells are more resistant to amphotericin B- and caspofungin-induced programmed cell death than their blastospore counterparts. Finally, our genetic data suggests that this phenomenon is mediated by a protective mechanism involving the yeast metacaspase, *MCA1*.

## INTRODUCTION

The budding yeast *Candida albicans* has emerged as one of the most significant fungal pathogens globally 1. As an opportunistic pathogen capable of life-threatening systemic infections, *C. albicans* poses a serious threat to immuno-compromised individuals, including AIDS patients, cancer patients undergoing chemotherapy, organ transplant recipients, and patients with advanced diabetes 2,3,4. Worldwide, invasive candidiasis is currently regarded as the fourth most common cause of nosocomial infections with an estimated mortality rate of 35% 5,6. Significantly, resistance to therapies traditionally used to treat candidiasis such as triazoles and amphotericin B is rising 7,8. Thus, there is a pressing need to develop more effective anti-fungal treatments.

There are a number of physiological characteristics of *C. albicans* known to contribute to its virulence. Most notably, the organism’s ability to undergo a reversible morphological transition from round, budding cells called ‘blastospores,’ to elongated cells attached end-to-end, called ‘filaments,’ is linked to its ability to infect a host: cells unable to become filamentous or vice versa have been shown to be avirulent in mouse and *C. elegans* models 9,10,11,12,13,14,15,16,17,18. The process by which *C. albicans* undergoes the transition from blastospores to filaments is known as ‘filamentation’. Within the filamentous form, we further individuate two distinct cellular morphologies. Pseudo-hyphal cells are attached end-to-end, exhibit constrictions at the septa, and have an elongated cell wall, while true hyphal cells of *C. albicans* are distinguished by the emergence of small cellular protrusions called ‘germ tubes’. While a recent study has shown that virulence can be decoupled from cell type in *C. albicans*, the connection between cell type and pathogenicity remains an important one 19.

Interestingly, there is growing evidence to support the claim that the drugs commonly used to treat patients suffering from *C. albicans* infections, induce cell death 20,21. Specifically, *C. albicans* cells cultured in media containing the common anti-fungal drugs, amphotericin B (AMB) and caspofungin (CAS), undergo an apoptotic-like programmed cell death 22,23,24,25. Programmed cell death is a cell suicide program that is essential for homeostasis, development, and disease prevention in many multi-cellular organisms 26,27,28,29. When it occurs in yeast, programmed cell death is accompanied by the nicking of DNA, the accumulation of reactive oxygen species (ROS), and the intracellular activation of the fungal caspases 30,31,32,33,34,35,36,37.

In multicellular organisms, the response to programmed cell death is cell-type specific, and the rate of cell death varies widely from tissue to tissue and cell-type to cell-type within the plant or animal 26. In this paper, we provide evidence that suggests that programmed cell death is also cell-type specific in yeast: filamentous *Candida* cells are more resistant to amphotericin B- and caspofungin-induced programmed cell death than their blastospore counterparts. Finally, our genetic data suggests that this phenomenon is mediated by a mechanism involving the yeast metacaspase *MCA1*.

## RESULTS AND DISCUSSION

**Figure 1:**
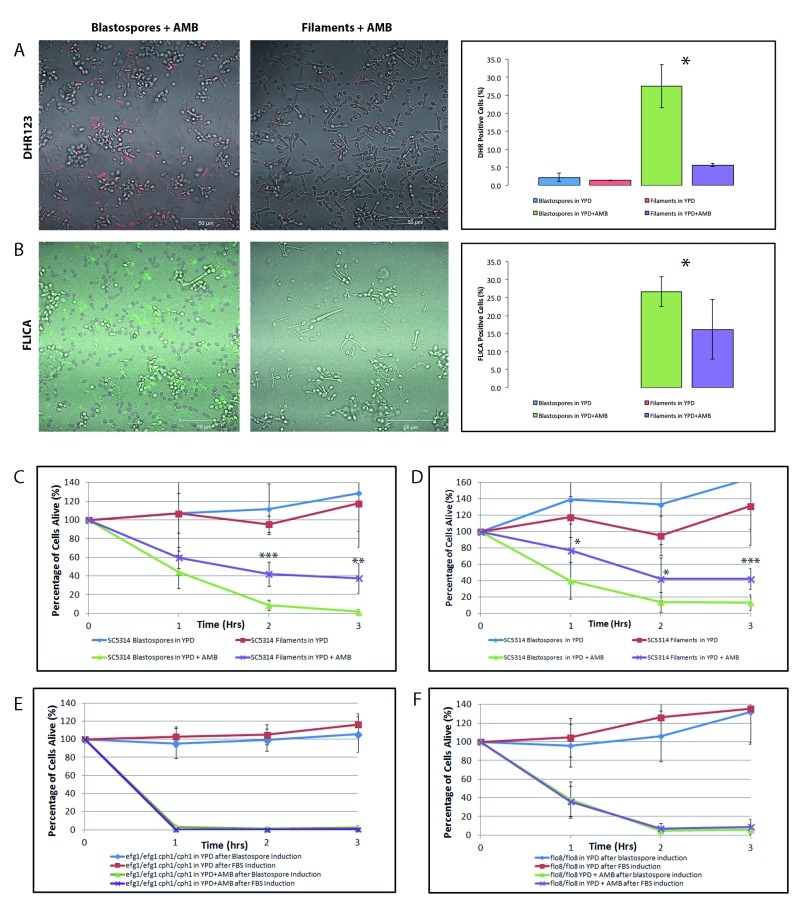
FIGURE 1: Filamentous *C. albicans* cells are more resistant than blastospores to AMB-induced programmed cell death. Exposure to amphotericin B leads to the generation of reactive oxygen species (ROS) and to caspase activation in *C. albicans* cells. Representative confocal scanning laser fluorescence images of wild-type SC5314 *C. albicans* cells treated with 8 μg/ml AMB for 3 hours in YPD. Staining with dihydrorhodamine 123 (DHR123) confirms the presence of ROS **(A)** and with the FLICA assay for activation of intracellular caspases **(B)**. Error bars indicate standard deviations for trials with at least three independent cultures, where at least 300 cells were counted for each trial. No FLICA positive cells were observed in the no drug controls. A single asterisk indicates statistical significance (p < 0.05) as compared to treated controls. Statistical significance was determined with the unpaired Student’s t-test. Scale bar: 50 μm. Viability curves compare survival of the following cells exposed to AMB: **(C)** wild type blastospores and wild type filaments induced using 10% FBS; **(D)** wild type blastospores and wild type filaments induced using 0.5 g/l GlcNAc; **(E)**ΔΔ*efg1/efg1 cph1/cph1* cells in YPD and ΔΔ*efg1/efg1 cph1/cph1* cells following filamentous induction in YPD + 10% FBS, and **(F)**ΔΔ*flo8/flo8* cells in YPD and ΔΔ*flo8/flo8* cells following filamentous induction in YPD + 10% FBS. Note that after 3 hr, cells cultured in rich media without any drugs were able to grow and to divide, hence the relative viability levels that are greater than 100%. Error bars indicate standard deviations for trials with at least three independent cultures. A single, double, and triple asterisk indicates a significance of p < 0.05, p < 0.005, and p < 0.0005, respectively, as compared to treated controls. Statistical significance was determined with the unpaired Student’s t-test.

In recent years, it has become evident that programmed cell death occurs in unicellular organisms. For example, in the pathogenic fungus *Candida albicans* exposure to acetic acid, hydrogen peroxide, AMB, CAS, and farnesol leads to cell death accompanied by hallmark features of mammalian programmed cell death 22,23,24,36,38. In multicellular organisms, the response to programmed cell death is cell-type specific, and the rate of cell death varies widely from tissue to tissue and cell type to cell type within the plant or animal 26. To determine whether or not different forms of yeast respond differently to stimuli that induce programmed cell death, we first investigated whether or not filamentous cells manifest the markers of programmed cell death when they are cultured in media containing AMB. In this study, the clinical isolate SC5314—the parent of strains widely used for molecular analysis—was used as the wild type strain 39. Briefly, overnight cultures of wild type cells in YPD were resuspended in YPD or YPD containing 10% fetal bovine serum (YPD+FBS) to obtain either blastospores or hyphal cells respectively (Supplemental Figure 1) 10,11,12. These cells were then resuspended in YPD containing 8 µg/ml AMB for 3 hours. Dihydrorhodamine 123 and FLICA staining confirmed that both these AMB-treated blastospores and filamentous cells accumulated ROS and activated caspases, respectively—two classic markers of programmed cell death – and were undergoing cell death as revealed by staining with propidium iodide (Figure 1). With both markers, however, there were fewer marker-positive filamentous cells as compared to blastospore controls, suggesting that the former cell type was more resistant to AMB.

Next, we compared the viability of wild-type *Candida albicans* cells in the blastospore and filamentous forms when cultured in media containing 8 µg/ml AMB with control cultures grown in YPD alone. Clonogenic survival assays are routinely used to assay programmed cell death in yeast 10,23,24,40,41. As shown in Figure 1C, hyphal cells had a higher viability when cultured in media containing AMB than their blastospore counterparts (p < 0.005). This data suggests that filamentation protects *Candida* cells from AMB-induced programmed cell death and that this type of programmed cell death is cell-type specific in yeast.

However, because hyphae were induced by culturing blastospores in media containing FBS 11, it is possible that the differences in clonogenic survival rate could be attributed to culture conditions—namely, the presence of FBS—rather than to filamentation. To rule out this alternative explanation for our observations, we repeated our assays with a filamentation induction protocol that used N-acetylglucosamine (GlcNAc) instead of FBS 42,43. As shown in Figure 1D, GlcNAc-induced filamentous cells were also more resistant than their blastospore counterparts to AMB-induced cell death. Still, it could be argued that the difference in survival rate observed between the two cell types was only due to the variable presence of either FBS or GlcNAc. To respond to this concern, we repeated our experiments with Can36, a SC5314-derived mutant yeast strain lacking *CPH1* and *EFG1*, two putative transcription factors necessary for filamentation in *Candida*^12^. As expected, this strain was unable to undergo filamentation in media containing 10% FBS (Supplemental Figure 1). However, as shown in Figure 1E, the viability of the ΔΔ*cph1/cph1 efg1/efg1* mutant yeast cells cultured in FBS and exposed to AMB was indistinguishable from that of mutant yeast cells cultured in media with AMB alone. Finally, we repeated our assay a fourth time with *CCF3*, a SC5314-derived ΔΔ*flo8/flo8* strain that is also unable to undergo filamentation when cultured in FBS 10. Again, this non-filamentous mutant was unable to survive when cultured in the presence of AMB regardless of whether or not it was first cultured in the presence of FBS [Figure 1F]. Complementation of the ΔΔ*flo8/flo8* strain confirmed that this phenotype, along with the inability to undergo filamentation, are both dependent upon the null ΔΔ*flo8/flo8* mutation as others had previously shown 10. Thus, we conclude that the resistance pattern noted in both non-filamentous mutants is not related to secondary effects of the mutations distinct from their inability to undergo filamentation, and that FBS itself is unable to protect yeast cells from AMB-induced programmed cell death. Together, these experiments suggest that filamentation protects yeast cells against AMB-induced programmed cell death.

**Figure 2:**
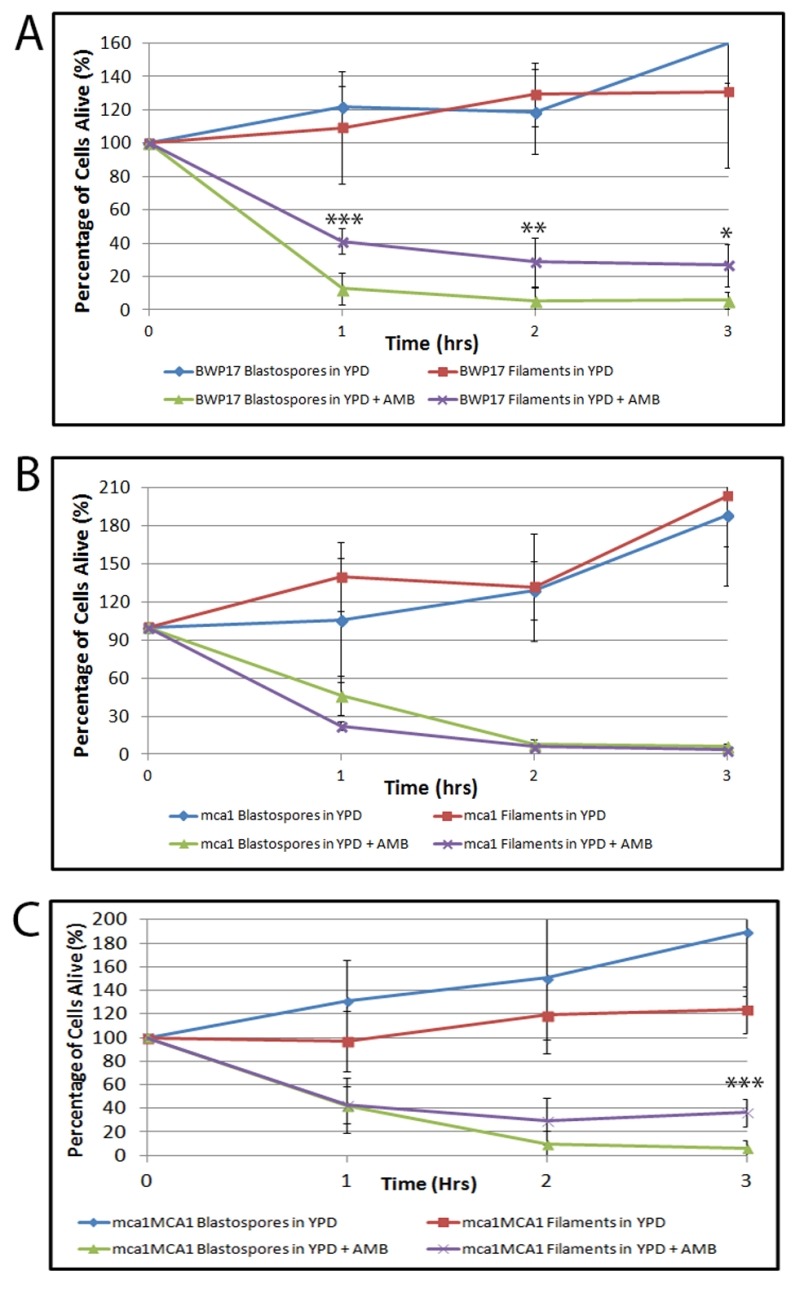
FIGURE 2: Filamentous *C. albicans* cells are more resistant than blastospores to AMB-induced programmed cell death in an *MCA1*-dependent manner. Viability curves compare survival of the following cells exposed to AMB: **(A)** wild type (BWP17) blastospores and BWP17 filaments induced using 10% FBS; **(B)** BWP17-derived ΔΔ*mca1/mca1* blastospores and ΔΔ*mca1/mca1* filaments induced using 10% FBS; and **(C)**ΔΔ*mca1/mca1::MCA1* blastospores and ΔΔ*mca1/mca1::MCA1* filaments induced using 10% FBS. Error bars indicate standard deviations for trials with at least three independent cultures. Note that after 3 hr, cells cultured in rich media without any drugs were able to grow and to divide, hence the relative viability levels that are greater than 100%. A single, double, and triple asterisk indicates statistical significance of p < 0.05, p < 0.005, and p < 0.0005, respectively, as compared to treated controls. Statistical significance was determined with the unpaired Student’s t-test.

To investigate the mechanism behind this anti-cell death phenomenon, we decided to focus on the yeast metacaspase, *MCA1*, a homolog of the mammalian caspases linked to apoptosis in metazoans. The *MCA1* homolog in *S. cerevisae*, *YCA1*, has been implicated in programmed cell death: mutants lacking *YCA1* in *S. cerevisae* exhibit lower levels of intracellular caspase activation and significantly decreased levels of programmed cell death when exposed to hyposomatic stress 32,44. We compared the survival rate of the wildtype BWP17 blastospores and filaments with their BWP17-derived ΔΔ*mca1/mca1* mutant counterparts. Wildtype and all *mca1* mutants were able to undergo filamentation when exposed to 10% FBS (Supplemental Figure 2). As shown in Figure 2, ΔΔ*mca1/mca1 *blastospores and hyphal cells had indistinguishable survival rates when cultured in media containing AMB. This data suggests that *MCA1* is involved in the resistance of filamentous cells to AMB-induced programmed cell death. Complementation of the null ΔΔ*mca1/mca1* mutant restored the original difference in viability that we had observed between blastospore and hyphal cells cultured in AMB-containing media, suggesting that the original ΔΔ*mca1/mca1* phenotype could be linked to the original loss-of-function mutation in *MCA1*. In sum, our data suggests that filamentation protects *C. albicans* cells from AMB induced cell death and that this phenotype is dependent upon the yeast metacaspase, *MCA1*. Given that *MCA1* has previously been thought to have a pro-death function, it is not yet clear how Mca1p functions in this protective capacity in filamentous cells. However, it is intriguing that several recent papers have revealed that the Mca1p homolog has a non-death role in *S. cerevisae* and possibly, in *C. albicans* as well 36,45,46,47,48,49.

**Figure 3:**
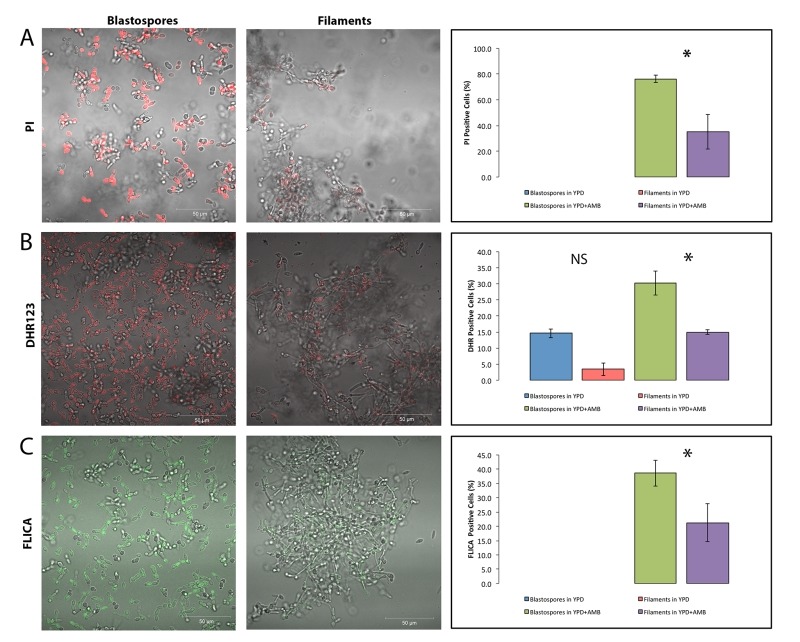
FIGURE 3: Filamentous *C. albicans* cells are more resistant than blastospores to caspofungin-induced programmed cell death. Representative confocal scanning laser fluorescence images of wild-type SC5314 C. albicans cells treated with 0.05 μg/ml caspofungin for 3 hours in YPD. Propidium iodide stains dead cells, dihydrorhodamine 123 (DHR123) indicates the presence of reactive oxygen species (ROS), and the FLICA assay stains for cells with activated intracellular caspases. No PI or FLICA positive cells were observed in the no drug controls. Error bars indicate standard deviations for trials with at least three independent cultures, where at least 300 cells were counted for each trial. A single asterisk indicates statistical significance (p < 0.05) as compared to treated controls. Statistical significance was determined with the unpaired Student’s t-test.

Finally, we wanted to determine if filamentation protected *Candida* cells from another anti-fungal drug known to induce programmed cell death. Thus, we compared the viability of blastospores and hyphal cells in media containing 0.05 µg/ml caspofungin (CAS), an echinocandin known to trigger cell death, in *Candida albicans*^22^,^23^. As shown in Figure 3, filamentation also appears to protect yeast cells from CAS-induced cell death suggesting the protective effects of filamentation may be a general phenomenon in *Candida albicans*. Watamoto *et al*. have proposed that filamentous *Candida* cells are resistant to AMB and to nystatin because they are able to form biofilms 17,50. In light of our findings, we also propose that planktonic hyphal cells may in themselves be relatively more resilient to these drugs—and possibly other anti-fungal drugs as well—because of their heightened resistance to programmed cell death.

## MATERIALS AND METHODS

### Media and Growth Conditions

*C. albicans* cells were grown in yeast extract/peptone/dextrose broth (YPD) made according to standard recipes 51. Cells were inoculated from single colonies growing on YPD plates into 20 ml YPD and grown under shaking at 30°C until the culture attained an OD_600_ value of 2.00 A. Once the culture had reached OD_600_≈2.0 A, cells were harvested and then resuspended in fresh media at a concentration of 3x10^7^ cells/ml (OD_600_≈1.26 A). For blastospore induction, cells from the original culture were resuspended in fresh YPD, transferred to a sterile flask, and then grown under shaking at 30°C for 3 hours. For hyphal induction, harvested cells were resuspended in either YPD + 10% fetal bovine serum (HyClone) pre-warmed to 37°C (YPD + FBS) or YPD + N-acetylglucosamine at a concentration of 0.5 g/l GlcNAc (Sigma-Aldrich; YPD+GlcNAc), transferred into a fresh flask, and placed in an incubator with shaking at 37°C for 3 hours 11,12,42,43,52.

### Viability Assays

Blastospores and hyphal cells were harvested and resuspended at a concentration of 1x10^7^ cells/ml in fresh YPD, and placed in 15 ml conical tubes. Cells were then exposed to AMB (Sigma) at a concentration of 5 µg/ml or 8 µg/ml (from a 1 mg/ml stock in dimethyl sulfoxide) for 3 hours, with shaking, at 25°C 24. At t=0, 1, 2, and 3 hours of AMB exposure, serial dilutions of the cell cultures were done on YPD plates. The plates were then placed in a 30°C incubator for 24 to 48 hours, or until single colonies were distinguishable. Colonies for each time point were counted and then compared as a percentage of the number of colonies that formed on the t=0 plate. For each time point, three independent cultures were tested. Notably, we confirmed our clonogenic assays by directly visualizing dead filamentous cells using propidum iodide (50 μg/ml) and then counting them with a Zeiss LSM700 fluorescent microscope. For the experiments with caspofungin, blastospores and filaments were cultured in the drug at a concentration of 0.05 μg/ml (from a 1 mg/ml stock in dimethyl sulfoxide) for 3 hours, with shaking, at 25°C. The viability of the cells was determined by culturing them in propidium iodide (50 μg/ml) and then counting them visually with a Zeiss LSM700 fluorescent microscope. Again, three independent cultures were tested, and at least 300 cells were counted for each determination. Statistical significance for all experiments was determined with the unpaired Student’s t-test.

### *In Vivo* Detection of ROS Accumulation and Caspase Activation

Intracellular ROS accumulation was examined after treatment with AMB or caspofungin using 5 μg/ml of dihydrorhodamine 123 (DH123; Sigma Aldrich) 24. Activated caspases were detected in *C. albicans* cells after treatment with AMB or CAS using a FLICA apoptosis detection kit (ImmunoChemistry Technologies, LLC) according to the manufacturer’s specifications 38. After exposure to either DHR123 or the FLICA reagent, *C. albicans* cells were harvested and examined using a Zeiss 700 Confocal Laser Scanning Microscope.

## SUPPLEMENTAL MATERIAL

Click here for supplemental data file.

All supplemental data for this article are also available online at http://microbialcell.com/researcharticles/filamentation-protects-candida-albicans-from-amphotericin-b-induced-programmed-cell-death-via-a-mechanism-involving-the-yeast-metacaspase-mca1/.

## References

[1] Guinea J (2014). Global trends in the distribution of Candida species causing
candidemia.. Clin Microbiol Infec.

[2] Cassone A, Cauda R (2012). Candida and candidiasis in HIV-infected patients: where
commensalism, opportunistic behavior and frank pathogenicity lose their
borders.. AIDS.

[3] Grossi PA (2009). Clinical aspects of invasive candidiasis in solid organ
transplant recipients.. Drugs.

[4] Sensoy G, Belet N (2011). Invasive Candida infections in solid organ transplant recipient
children.. Expert Rev Anti Infect Ther.

[5] Pfaller M, Neofytos D, Diekema D, Azie N, Meier-Kriesche HU, Quan SP, Horn D (2012). Epidemiology and outcomes of candidemia in 3648 patients: data
from the Prospective Antifungal Therapy (PATH Alliance(R)) registry,
2004-2008.. Diagn Microbiol Infect Dis.

[6] Mikulska M, Del Bono V, Ratto S, Viscoli C (2012). Occurrence, presentation and treatment of
candidemia.. Expert Rev Clin Immunol.

[7] Huang M, Kao KC (2012). Population dynamics and the evolution of antifungal drug
resistance in Candida albicans.. FEMS Microbiol Lett.

[8] Pfaller MA (2012). Antifungal drug resistance: mechanisms, epidemiology, and
consequences for treatment.. Am J Med.

[9] Brennan M, Thomas DY, Whiteway M, Kavanagh K (2002). Correlation between virulence of Candida albicans mutants in mice
and Galleria mellonella larvae.. FEMS Immunol Med Microbiol.

[10] Cao F, Lane S, Raniga PP, Lu Y, Zhou Z, Ramon K, Chen J, Liu H (2006). The Flo8 transcription factor is essential for hyphal development
and virulence in Candida albicans.. Mol Biol Cell.

[11] Kadosh D, Johnson AD (2005). Induction of the Candida albicans filamentous growth program by
relief of transcriptional repression: a genome-wide
analysis.. Mol Biol Cell.

[12] Lo HJ, Kohler JR, DiDomenico B, Loebenberg D, Cacciapuoti A, Fink GR (1997). Nonfilamentous C. albicans mutants are avirulent.. Cell.

[13] Pukkila-Worley R, Peleg AY, Tampakakis E, Mylonakis E (2009). Role of filamentation in Galleria mellonella killing by Candida
albicans.. Eukaryot Cell.

[14] Staib P, Binder A, Kretschmar M, Nichterlein T, Schroppel K, Morschhauser J (2004). Tec1p-independent activation of a hypha-associated Candida
albicans virulence gene during infection.. Infect Immun.

[15] Fuchs BB, Eby J, Nobile CJ, El Khoury JB, Mitchell AP, Mylonakis E (2010). Role of filamentation in Galleria mellonella killing by Candida
albicans.. Microbes Infect.

[16] Laforet L, Moreno I, Sanches-Fresneda R, Martinez-Esparza M, Martinez JP, Arguelles JC, de Groot PW, Valentin-Gomez E (2011). Pga26 mediates filamentation and biofilm formation and is
required for virulence in Candida albicans.. FEMS Yeast Res.

[17] Watamoto T, Samaranayake LP, Egusa H, Yatani H, Samaranayke YH, Seneviratne CJ (2010). Susceptibility of Candida albicans filamentation-defective
mutants to clinical biocides.. J Hosp Infect.

[18] O‘Meara TR, Veri AO, Ketela T, Jiang B, Roemer T, Cowen LE (2015). Global analysis of fungal morphology exposes mechanisms of host
cell escape.. Nat Commun.

[19] Noble SM, French S, Kohn LA, Chen V, Johnson AD (2010). Systematic screens of a Candida albicans homozygous deletion
library decouple morphogenetic switching and pathogenicity.. Nat Genet.

[20] Ramsdale M (2008). Programmed cell death in pathogenic fungi.. Biochem Biophys acta.

[21] Almeida B, Silva A, Mesquita A, Sampaio-Marques B, Rodrigues F, Ludovico P (2008). Drug -induced apoptosis in yeast.. Biochem Biophys acta.

[22] Chin C, Donaghey F, Helming K, McCarthy M, Rogers S, Austriaco N (2014). Deletion of AIF1 but not of YCA1/MCA1 protects Saccharomyces
cerevisiae and Candida albicans cells from caspofungin-induced programmed
cell death.. Microb Cell.

[23] Hao B, Cheng S, Clancy CJ, Nguyen MH (2013). Caspofungin kills Candida albicans by causing both cellular
apoptosis and necrosis.. Antimicrob Agents Chemother.

[24] Phillips AJ, Sudbery I, Ramsdale M (2003). Caspofungin Apoptosis induced by environmental stresses and
amphotericin B in Candida albicans.. Proc Natl Acad Sci U S A.

[25] Lin SJ, Austriaco N (2014). Aging and cell death in the other yeasts, Schizosaccharomyces
pombe and Candida albicans.. FEMS Yeast Res.

[26] Kerr JF, Wyllie AH, Currie AR (1972). Apoptosis: a basic biological phenomenon with wide-ranging
implications in tissue kinetics.. Br J Cancer.

[27] Ameisen JC (2002). On the origin, evolution, and nature of programmed cell death: a
timeline of four billion years.. Cell Death Differ.

[28] Zmasek CM, Godzik A (2013). Evolution of the animal apoptosis network.. Cold Spring Harb Perspect Biol.

[29] Teng X, Hardwick JM (2015). Cell death in genome evolution.. Semin Cell Dev Biol.

[30] Carmona-Gutierrez D, Eisenberg T, Buttner S, Meisinger C, Kroemer G, Madeo F (2010). Apoptosis in yeast: triggers, pathways,
subroutines.. Cell Death Differ.

[31] Liang Q, Li W, Zhou B (2008). Caspase-independent apoptosis in yeast.. Biochim Biophys Acta.

[32] Madeo F, Carmona-Gutierrez D, Ring J, Buttner S, Eisenberg T, Kroemer G (2009). Caspase-dependent and caspase-independent cell death pathways in
yeast.. Biochem Biophys Res Commun.

[33] Munoz AJ, Wanichthanarak  K, Meza E, Petranovix D (2012). Systems biology of yeast cell death.. FEMS Yeast Res.

[34] Wong AH, Yan  C, Shi Y (2012). Crystal structure of the yeast metacaspase Yca1.. J Biol Chem.

[35] Wilkinson D, Ramsdale  M (2011). Proteases and caspase-like activity in the yeast Saccharomyces
cerevisiae.. Biochem Soc Trans.

[36] Leger T, Garcia  C, Ounissi  M, Lelandais  G, Camadro  JM (2015). The metacaspase (Mca1p) has a dual role in farnesol-induced
apoptosis in Candida albicans.. Mol Cell Proteomics.

[37] Strich R (2015). Programmed Cell Death Initiation and Execution in Budding
Yeast.. Genetics.

[38] Shirtliff ME, Krom BP, Meijering RA, Peters BM, Zhu J, Scheper MA, Harris ML, Jabra-Rizk MA (2009). Farnesol-induced apoptosis in Candida albicans.. Antimicrob Agents Chemother.

[39] Fonzi WA, Irwin MY (1993). Isogenic strain construction and gene mapping in Candida
albicans.. Genetics.

[40] Aerts AM, Carmona-Gutierrez D, Lefevre S, Govaert G, Francois IE, Madeo F, Santos R, Cammue BP, Thevissen K (2009). The antifungal plant defensin RsAFP2 from radish induces
apoptosis in a metacaspase independent way in Candida
albicans.. FEBS Lett.

[41] Nguyen  KT, Ta P, Hoang BT, Cheng S, Hao B, Nguyen MH, Clancy CJ (2009). Anidulafungin is fungicidal and exerts a variety of
postantifungal effects against Candida albicans, C. glabrata, C.
parapsilosis, and C. krusei isolates.. Antimicrob Agents Chemother.

[42] Bauer J, Wendland J (2007). Candida albicans Sfl1 suppresses flocculation and
filamentation.. Eukaryot Cell.

[43] Hornby JM, Jensen EC, Lisec AD, Tasto JJ, Jahnke B, Shoemaker R, Dussault P, Nickerson KW (2001). Quorum sensing in the dimorphic fungus Candida albicans is
mediated by farnesol.. Appl Environ Microbiol.

[44] Mazzoni  C, Falcone C (2008). Caspase-dependent apoptosis in yeast.. Biochim Biophys Acta.

[45] Cao  Y, Huang S, Dai B, Zhu Z, Lu H, Dong L, Cao Y, Wang Y, Gao P, Chai Y, Jiang Y (2009). Candida albicans cells lacking CaMCA1-encoded metacaspase show
resistance to oxidative stress-induced death and change in energy
metabolism.. Fungal Genet Biol.

[46] Erhardt M, Wegrzyn RD, Deuerling E (2010). Extra N-terminal residues have a profound effect on the
aggregation properties of the potential yeast prion protein
Mca1.. PLoS One.

[47] Lee RE, Brunette S, Puente LG, Megeney LA (2010). Metacaspase Yca1 is required for clearance of insoluble protein
aggregates.. Proc Nat Acad Sci U S A.

[48] Shrestha A, Puente LG, Brunette S, Megeney LA (2013). The role of Yca1 in proteostasis. Yca1 regulates the composition
of the insoluble proteome.. J Proteomics.

[49] Hill SM, Hao X, Liu B, Nystrom T (2014). Life-span extension by a metacaspase in the yeast Saccharomyces
cerevisiae.. Science.

[50] Watamoto T, Samaranayake  LP, Jayatilake  JA, Egusa H, Yatani H, Seneviratne CJ (2009). Effect of filamentation and mode of growth on antifungal
susceptibility of Candida albicans.. Int J Antimicrob Agents.

[51] Burke DJ, Amberg DC, Strathern JN (2005). Methods in Yeast Genetics: A Cold Spring Harbor Laboratory
Course Manual.. Cold Spring Harbor Laboratory Press, Cold Spring Harbor, NY..

[52] Lee KL, Buckley HR, Campbell CC (1975). An amino acid liquid synthetic medium for the development of
mycelial and yeast forms of Candida Albicans.. Sabouraudia.

