# Supplementary material for: Filamentation protects Candida albicans from amphotericin B-induced programmed cell death via a mechanism involving the yeast metacaspase, MCA1
Source: Microb Cell. 2016 Apr 25;3(7):285–92. doi: 10.15698/mic2016.07.512 (PMC5036395; doi:10.15698/mic2016.07.512)
Supplement: Supplementary file 1 [file mic-03-285-s01.pdf]

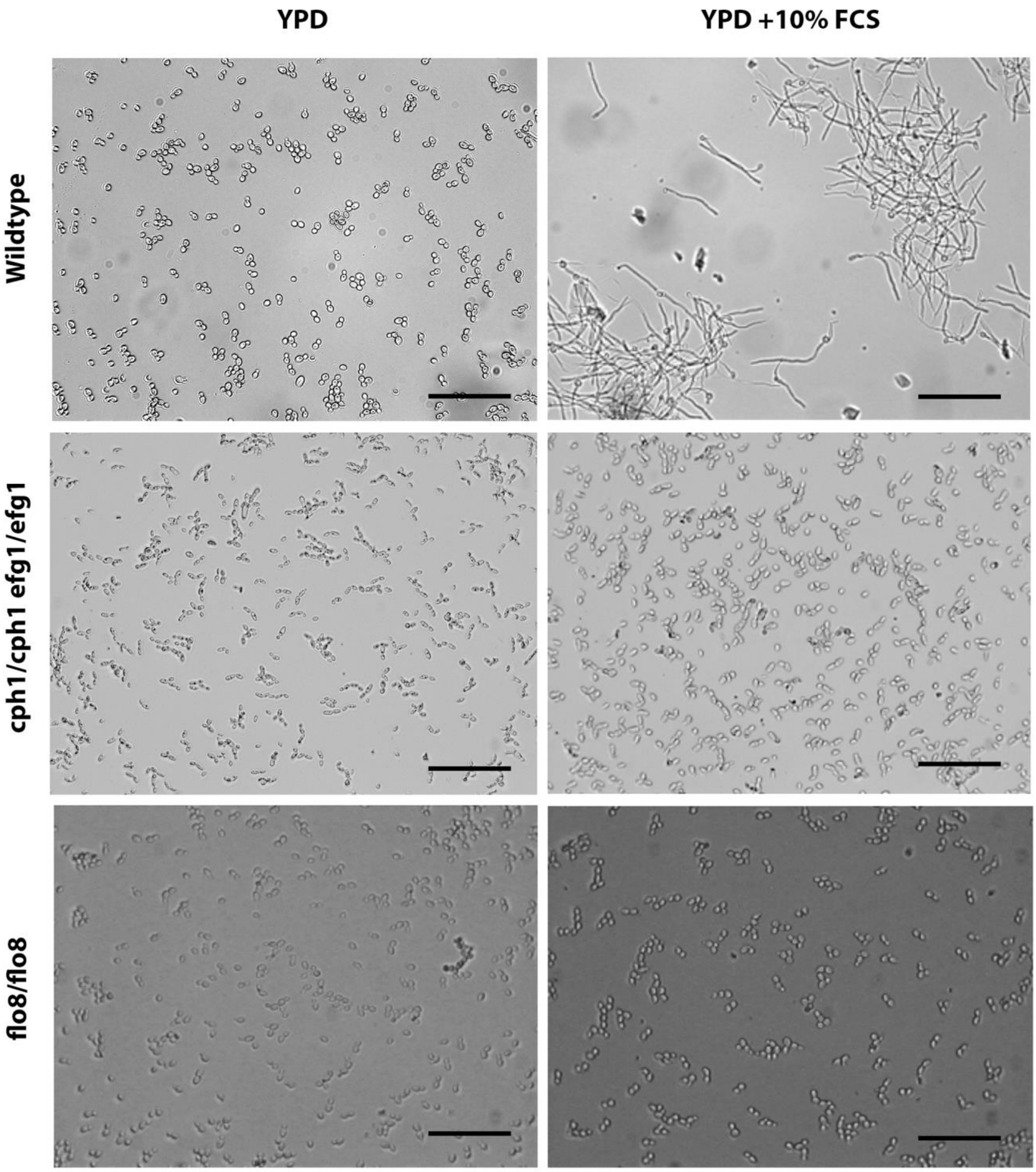

SUPPLEMENTAL FIGURE 1: Fetal calf serum (FCS) induces filamentation in *C. albicans* wildtype cells but not in nonfilamentous mutant cells. Micrographs (200X magnification) of wild-type and of nonfilamentous mutants in the SC5314 strain background cultured in YPD and in YPD media supplemented with 10 % FCS for three hours at 37°C. Scale bar: 50 µm.

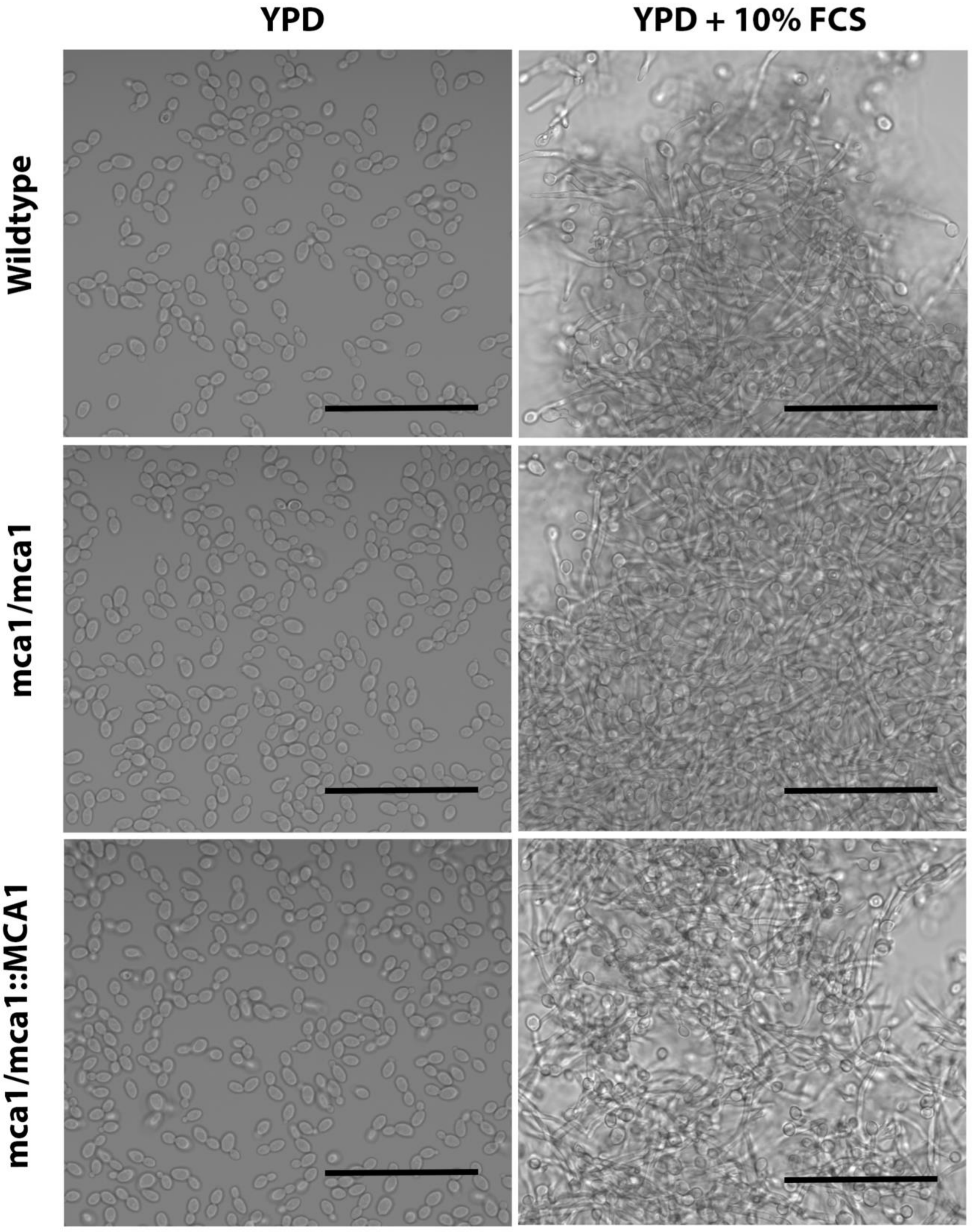

SUPPLEMENTAL FIGURE 2: Fetal calf serum (FCS) induces filamentation both in *C. albicans* wildtype cells and *mca1* mutant cells. Micrographs of wild-type and of *mca1* mutants in the BWP17 strain background cultured in YPD and in YPD media supplemented with 10 % FCS for three hours at 37°C. Scale bar: 50 µm.
